# Multifunctional single beam acoustic tweezer for non-invasive cell/organism manipulation and tissue imaging

**DOI:** 10.1038/srep37554

**Published:** 2016-11-22

**Authors:** Kwok Ho Lam, Ying Li, Yang Li, Hae Gyun Lim, Qifa Zhou, Koping Kirk Shung

**Affiliations:** 1 Department of Electrical Engineering, The Hong Kong Polytechnic University, Hong Kong; 2 NIH Transducer Resource Center and Department of Biomedical Engineering, University of Southern California, Los Angeles, CA 90089, USA

## Abstract

Non-contact precise manipulation of single microparticles, cells, and organisms has attracted considerable interest in biophysics and biomedical engineering. Similar to optical tweezers, acoustic tweezers have been proposed to be capable of manipulating microparticles and even cells. Although there have been concerted efforts to develop tools for non-contact manipulation, no alternative to complex, unifunctional tweezer has yet been found. Here we report a simple, low-cost, multifunctional single beam acoustic tweezer (SBAT) that is capable of manipulating an individual micrometer scale non-spherical cell at Rayleigh regime and even a single millimeter scale organism at Mie regime, and imaging tissue as well. We experimentally demonstrate that the SBAT with an ultralow f-number (f# = focal length/aperture size) could manipulate an individual red blood cell and a single 1.6 mm-diameter fertilized Zebrafish egg, respectively. Besides, *in vitro* rat aorta images were collected successfully at dynamic foci in which the lumen and the outer surface of the aorta could be clearly seen. With the ultralow f-number, the SBAT offers the combination of large acoustic radiation force and narrow beam width, leading to strong trapping and high-resolution imaging capabilities. These attributes enable the feasibility of using a single acoustic device to perform non-invasive multi-functions simultaneously for biomedical and biophysical applications.

With highly focused laser beams, optical tweezers^1^,^2^ have widely been used to study biophysical properties of cells and molecules^3^,^4^,^5^. Although optical tweezers exhibit an absolute advantage on manipulating tiny objects with high resolution due to the nature of light, they are commonly limited to optical purified samples. Besides, the targeted biological samples would easily be damaged by the highly focused laser beam-induced local heat. In addition, the force of optical tweezers is usually too small to manipulate relatively large particles or cells. Compared to the optical tweezers, the simpler setup of acoustic devices would be with lower cost. The most key advantage of using acoustic devices is that the biological samples are less likely to be damaged by the acoustic energy, which has been reported in the previous study^6^,^7^,^8^. Thus, the acoustic approach is considered to be an alternative non-invasive way to offer the capability of manipulation for biomedical and biophysical applications.

In recent years, different types of acoustic devices have been reported for manipulation purposes. However, most of them, such as standing wave^9^ and Bessel beams^10^, are complicated in configurations, which can only be operated either at Rayleigh (the trapped object size is smaller than the wavelength) or Mie regime (the trapped object size is larger than the wavelength) or groups of particles. Recently, acoustic systems based on surface acoustic wave^11^ have also been reported to be capable of on-chip manipulating both the cells and even C. elegans. Among those devices employed with different techniques, a single beam acoustic tweezer (SBAT)^12^ with the simplest configuration has been shown to be capable of manipulating a single microparticle in both theoretical and experimental approaches^13^,^14^,^15^ (see the working mechanism in Supplementary Figure 1). Similar to the optical tweezers, a tightly focused ultrasound microbeam produced by the SBAT could manipulate the object at Rayleigh^16^ and Mie^13^,^17^,^18^ regimes. Previously, a single polystyrene microsphere of 5 *μ*m diameter was manipulated by highly sensitive ultrahigh frequency (~200 MHz) SBATs within a range of hundreds of micrometers in distilled water^13^. Although previous studies suggested that the SBAT is capable of manipulating particles at the cellular level and even spherical cells^13^,^17^, it still has shown no demonstration on manipulating non-spherical objects owing to the gentle intensity gradient around the focus of the microbeam.

Based on the pre-focused single-element ultrasonic transducer design^19^, the practical performance of SBATs could be tailor made by changing the intensity gradient of the microbeam for specific applications. More specifically, the frequency and f-number (*f*# = focal length/aperture size) of SBATs mainly determine the beam width and acoustic radiation force. As the beam width increases and the beam intensity at focus decreases while increasing the *f*# (see the simulated results in Supplementary Figure 2), in order to perform manipulation much efficiently, the *f*# of the device must be further reduced to allow the microbeam to form the steeper intensity gradient around its focus. In this work, we designed a versatile SBAT with an ultralow *f*# that is capable of manipulating a single non-spherical cell and even a small organism at different regimes. In general, high lateral resolution could be obtained when the beam width of ultrasound is narrow^20^. With the ultralow *f*#, the SBAT would exhibit relatively narrow beam width, resulting in high imaging resolution. Thus, besides the outstanding manipulation capability, the SBAT also exhibits the superior imaging performance.

Figure 1 shows an acoustic tweezing experimental setup and an example of an individual red blood cell (RBC) manipulation using a 60–MHz SBAT. The *f*# of the SBAT is 0.6 that approaches the physical limit of the press-focused device. During the study, the SBAT will randomly target and acoustically manipulate the RBC suspended in Alsever’s solution (see Fig. 1a). In Fig. 1b, the bright circular shape is the projection of the SBAT. A red circle represents the single trapped RBC (~8 *μ*m diameter) while a yellow dot is given as a reference point to show the location change of the cell. The SBAT was driven with the optimal condition as follows: an excitation frequency of 60 MHz, a driving voltage of 20 V_p-p_, a duty cycle of 0.2%, and a pulse repetition frequency of 1 kHz. It is shown that the cell moves along with the SBAT movement. The individual cell manipulation is efficient, which would not be affected by the cells nearby (see Supplementary Video 1). It should be noted that this is the first time to manipulate an individual non-spherical cell using the acoustic microbeam device.

Although the optical tweezers exhibit the absolute advantage on manipulating tiny particles with high resolution, the force is too small to handle big objects. For SBATs, the design of smaller *f*# offers the steeper intensity gradient of the microbeam, resulting in larger acoustic radiation force. Using the identical (60–MHz *f*# 0.6) SBAT, even a single fish egg could be acoustically manipulated. Figure 2a shows that fertilized Zebrafish eggs are with the transparent appearance while a dead egg is with milky color. Compared to the cell, the Zebrafish egg with a diameter of 1.6 mm is much bigger. With a yellow dot as a location reference point as shown in Fig. 2b, it is obvious that the fertilized egg could be acoustically manipulated using the SBAT driven at its center frequency with a voltage of 32 V_p-p_, a duty cycle of 0.2% and a pulse repetition frequency of 1 kHz. Supplementary Video 2 shows that the fertilized egg with a live embryo does not move initially when the SBAT is off. After turning on the SBAT, the egg moves properly according to the SBAT movement. This is the biggest living organism manipulation performed with the SBAT.

To show the non-invasiveness of SBAT, we performed the viability test on the RBC under the exposure of SBAT with the same driving conditions in acoustic manipulation. Figure 3a shows the normalized mean viability of RBC before and after the exposure of SBAT at specific time intervals. It was found that the fluorescence of RBC was not significantly changed in 30 mins after the exposure of SBAT (*p*-value = 0.485, >0.01). Figure 3b illustrates that the RBC still emitted strong green fluorescence at 30 min, showing good viability under the experimental conditions in acoustic manipulation. The results suggest that the SBAT is of non-invasive and biocompatible.

Besides the manipulation studies, the biomedical imaging performance of the identical SBAT was evaluated. Figure 4 shows *in vitro* rat aorta tissue images collected at dynamic foci, where the SBAT was gradually moved closer to the sample. As shown in Fig. 4a, the lumen and the outer surface of the aorta could be clearly imaged near the focal point of SBAT. When the SBAT was moved closer to the sample, the upper part of the image was blurred with distortion, which is mainly attributed to the attenuated signal caused by the strong divergence of acoustic beam of the tightly focused device. By compounding sub-images of dynamic foci, a high quality B-mode/Depth-scan (B/D-scan)^21^ image can be reconstructed. With using proper windowing/weighting functions, near-focus segmentation from sub-images acquired at different device-to-sample distances can be compounded to achieve an image with large depth of view. Figure 4b shows the compounded image of rat aorta. Compared to the sub-images in Fig. 4a, the compounded image is uniform across the depth direction with enhanced contrast and smaller near field artifacts. The complete shape and boundary of the aorta can be clearly distinguished. The specific speckle pattern is attributed to the high-frequency tightly focused imaging^22^ and the scattering structure interrogated^23^. The dynamic range of the B/D-scan image is 50 dB, which is consistent to the dynamic range of the B-scans used for compounding. This method successfully extended the imaging depth of view while maintained the resolution and dynamic range of the B-scan images. Given the small speckle and high contrast of the image, the tissue imaging results do not only show the imaging capability of the SBAT, but also demonstrate the capability of the proposed method for studying tissue structures at the microscopic level.

The ultralow *f*# SBAT we developed is simple, low-cost, and versatile, which exhibits the outstanding acoustic manipulation capability as well as the superior imaging performance. With the *f*# of 0.6, the very steep intensity gradient of acoustic microbeam offers strong trapping capability such that the SBAT is capable of manipulating the individual non-spherical RBC and the single fertilized Zebrafish egg, respectively. Regarding the size of trapped objects, the SBAT could be operated at Rayleigh (RBC size of 8 *μ*m < wavelength of 25 *μ*m @ 60 MHz) and Mie (Zebrafish egg size of 1600 *μ*m ≫ wavelength of 25 *μ*m @ 60 MHz) regimes. Besides the acoustic manipulation, the identical SBAT is also capable of imaging tissues. With using the proposed method, *in vitro* rat aorta tissue image with large depth of view could be acquired. We anticipate this discovery will bring out possibilities that various functions can be performed by a multifunctional acoustic device for biomedical applications, such as manipulating-while-imaging for drug delivery or stimulating-while-imaging for cell characterization.

## Methods

### SBAT development

SBATs were designed by a Krimholtz, Leedom and Matthaei (KLM) model. The optimized thickness of the lithium niobate (LiNbO_3_) single crystal was ~45 *μ*m with an aperture size of 1.6 mm for a center frequency of 60 MHz. The first acoustic matching layer was a *λ*/4-thick silver epoxy made from a mixture of silver particles (Adrich Chem. Co., Milwaukee, WI) and Insulcast 501 epoxy (American Safety Technologies, Roseland, NJ), which was cast onto the negative side of the chrome/gold sputtered wafer. A conductive silver epoxy (E-Solder 3022, Von Roll Isola Inc., New Haven, CT) served as a backing layer, which was cast onto the positive side of the wafer. The acoustic stack with matching and backing layers was press-focused at a focal length of 1.0 mm so as to obtain a *f#* of ~0.6. A ~10 *μ*m-thick parylene layer was vapor-deposited on the front face of the SBAT to serve as the second matching layer as well as a protection layer. The SBAT was assembled in an SMA connector for further experiments.

### Acoustic tweezing experiments

**Figure 1:**
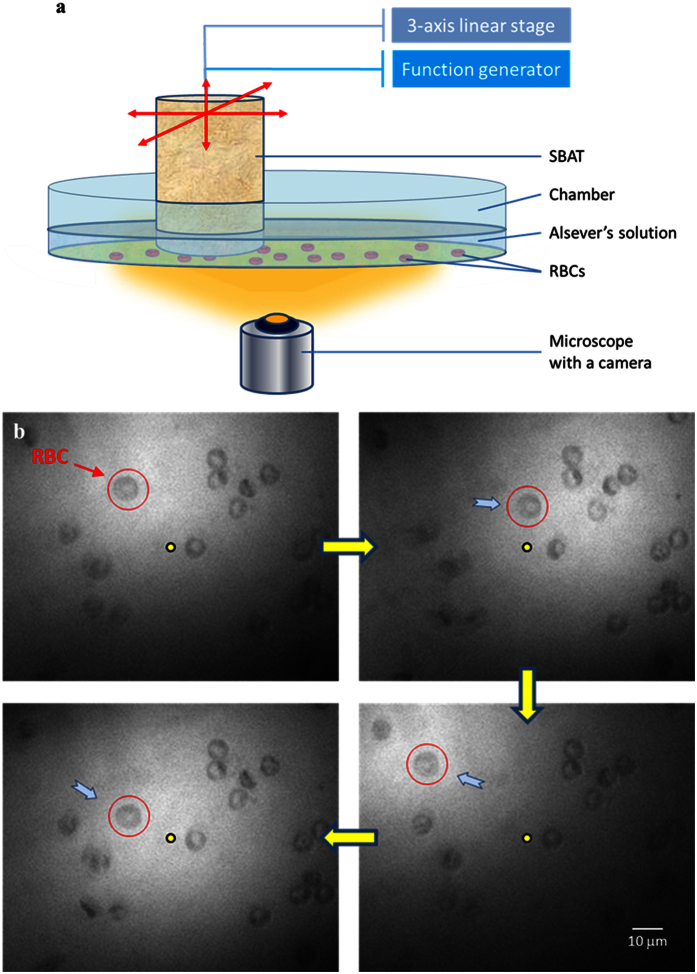
Red blood cell (RBC) manipulated acoustically using the 60-MHz *f*#0.6 SBAT. (**a**) Schematic diagram of acoustic tweezing experimental setup for RBC manipulation. (**b**) A single RBC (a red circle) was manipulated along the movement of the SBAT. The bright circular shape is the projection of the SBAT. A yellow dot is given as a reference point to show the location change of the cell while a blue arrow indicates the SBAT movement direction.

An acoustic tweezing experimental setup was built with a SBAT in a chamber of solution (see Fig. 1a). The solution was Alsever’s solution (A3551, Sigma-Aldrich, MO) for the red blood cell study while the solution was water provided by the Zebrafish egg supplier (Caroline Biological Supply Company) for the Zebrafish egg study. For the red blood cell experiment, ~4 *μ*l blood was drawn from a healthy donor and washed three times in 1 ml phosphate-buffered saline (PBS). Washed cells were diluted to 10^5^ cells/*μ*l in Alsever’s solution.

During the experiments, the objects were suspended in the solution. A single object was randomly targeted within the field of view of the microscope. The SBAT was mounted on a three-axis motorized stage (LMG26 T50 MM; OptoSigma, Santa Ana, CA) controlled by a customized LabVIEW program such that the SBAT was manipulated perpendicularly to the beam axis at the focal distance above an acoustically transparent mylar film. The SBAT was driven in a sinusoidal burst mode at optimal driving conditions with combinations of frequency, voltage, duty factor and pulse repetition frequency. The trapped motions of the object were recorded with a capturing frame rate of 10 frames/second via a CMOS camera (ORCA-Flash2.8, Hamamatsu, Japan) combined with a microscope (IX-71, Olympus, Japan). To show the capability of acoustic manipulation, the SBAT with the trapped object was moved in a random path by the motorized stages.

### Cell viability test

Fresh blood was drawn from a healthy donor and washed three times in phosphate-buffered saline (PBS). In each cell viability test, a membrane-permeable live-cell labeling dye, Calcein AM (In-vitrogen Corp., Grand Island, NY, USA), was prepared as a stock solution of 1 mM in dimethylsulfoxide stored at room temperature. 10 *μ*M of calcein-AM working solution (final concentration) was loaded into the cells. Fluorescence imaging of cells was performed before and after the exposure of SBAT with the highest driving power for acoustic manipulation (voltage = 32 V_p-p_, duty cycle = 0.2%, pulse repetition frequency = 1 kHz, exposure time = 30 s). The viability change of the cells was compared at specific time intervals (0, 10, 20 and 30 mins). For a statistical analysis, the mean and standard deviation of the fluorescence level at each time interval were obtained with a sample size of n = 10.

### Imaging experiment

A rat aorta of ~0.9 cm diameter was harvested by surgeon at the School of Medicine of the University of Southern California, which was stored in Eurocollins solution at 4 °C before the experiment. Images were acquired using an ultrasound biomicroscopy (UBM) setup. The SBAT was attached with a 3D motorized linear stage (Newport Corporation, CA) for scanning. Tissue samples were coupled using water. Excitation pulse and echo signal was generated and received by a commercial pulser receiver (JSR Ultrasonics, NY). The signal was digitized using a 2 GHz high-speed data acquisition card (Gage, IL). A Matlab (Mathwork, MA)-based controlling and imaging processing software was developed, in which the echo signal was filtered by a 4^th^ order Butterworth band-pass filter and the enveloped information was detected using Hilbert transform. During combining the sub-images, a triangular weighting function was used in which the weighting factor was 1 at the focus and linearly decreased to 0 at 100 *μ*m from the focus. A trapezoid weighting function was used for the images with the closest/farthest focus. The compounded image was then calculated by applying the corresponding weighting functions to post-filter the images.

### Study approval/Informed consent/Accordance

All experiments on live vertebrates were performed in accordance with protocols approved by the Institutional Animal Care and Use Committee at the University of Southern California.

## Additional Information

**How to cite this article**: Lam, K. H. *et al*. Multifunctional single beam acoustic tweezer for non-invasive cell/organism manipulation and tissue imaging. *Sci. Rep.* **6**, 37554; doi: 10.1038/srep37554 (2016).

**Publisher’s note:** Springer Nature remains neutral with regard to jurisdictional claims in published maps and institutional affiliations.

## Supplementary Material

Supplementary Information

Supplementary Video 1

Supplementary Video 2

## Figures and Tables

**Figure 2:**
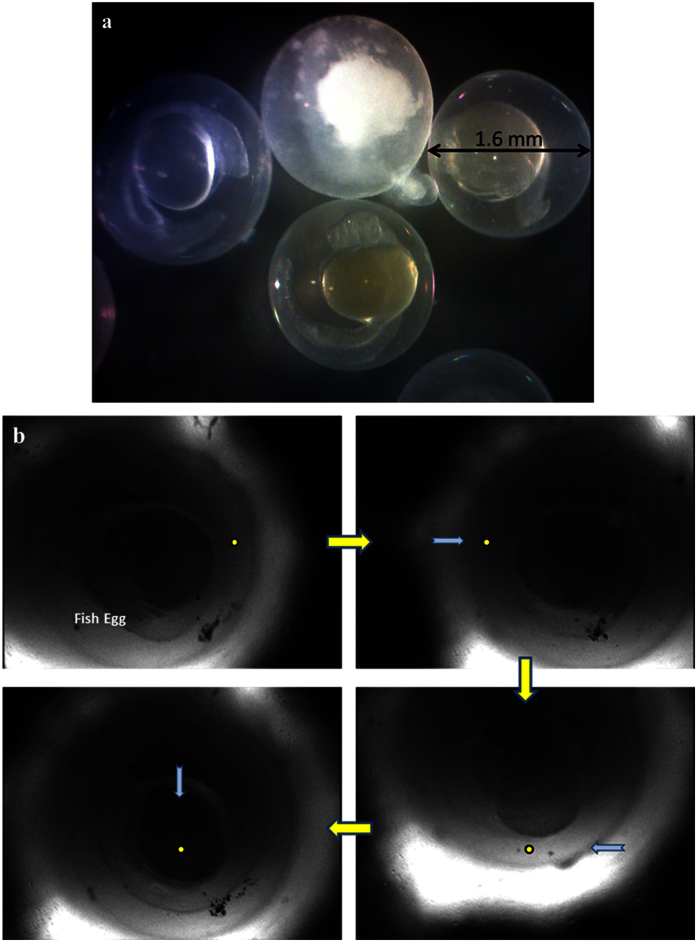
Zebrafish egg manipulated acoustically using the 60-MHz *f*#0.6 SBAT. (**a**) Fertilized Zebrafish eggs with a live embryo are in transparent appearance while a dead egg (on the top) is with milky color. The average diameter of Zebrafish eggs is 1.6 mm. (**b**) A single fertilized Zebrafish egg was acoustically manipulated along the SBAT movement. The bright circular shape is the projection of the SBAT. A yellow dot is given as a reference point to show the location change of the fish egg while a blue arrow indicates the SBAT movement direction.

**Figure 3:**
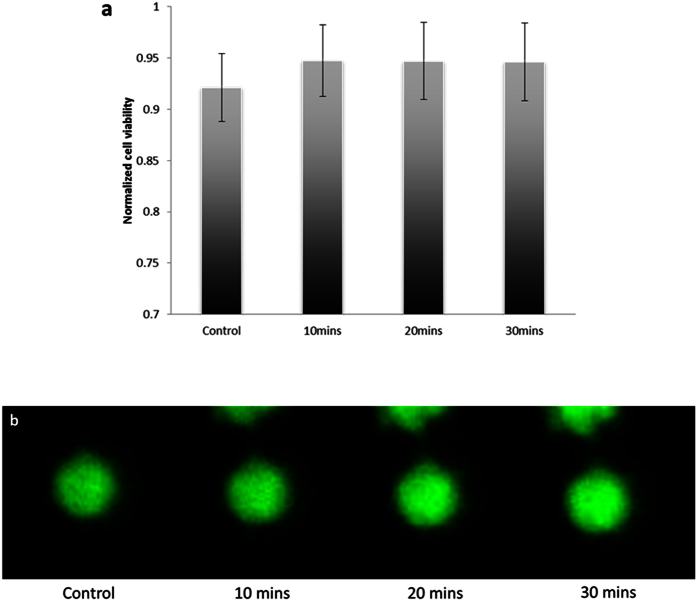
RBC viability before and after the exposure of SBAT with the driving conditions for acoustic manipulation (voltage = 32 Vp-p, duty cycle = 0.2%, pulse repetition frequency = 1 kHz, exposure time = 30 s). (**a**) Normalized cell viability at specific time intervals (0 (control), 10, 20 and 30 mins). (**b**) The corresponding representative fluorescence images. (Green fluorescence represents a viable cell).

**Figure 4:**
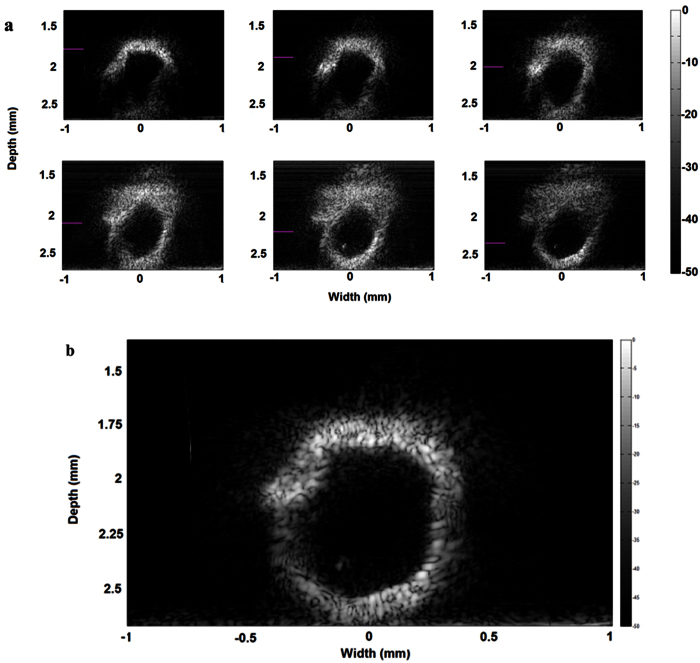
*In vitro* B/D-scan tissue images acquired by the 60-MHz *f*#0.6 SBAT. (**a**) Rat aorta sub-images collected (5 *μ*m lateral step) at different device-to-sample distances. The focus position is labeled with a purple bar. (**b**) The resultant aorta image, compounded with the sub-images (B/D-scan) shown in Fig. 3a, exhibits enhanced contrast and large depth of view.
