# Supplementary material for: Multifunctional single beam acoustic tweezer for non-invasive cell/organism manipulation and tissue imaging
Source: Sci Rep. 2016 Nov 22;6:37554. doi: 10.1038/srep37554 (PMC5118718; doi:10.1038/srep37554)
Supplement: Supplementary Information [file srep37554-s1.pdf]

Multifunctional single beam acoustic tweezer for non-invasive cell/organism manipulation and tissue imaging

Kwok Ho Lam[1], Ying Li[2], Yang Li[2], Hae Gyun Lim[2], Qifa Zhou[2], Koping Kirk Shung[2]

List of supplementary information file

Lam_EDS1.mov	Acoustic manipulation of an individual red blood cell
Lam_EDS2.mov	Acoustic manipulation of a fertilized Zebrafish egg

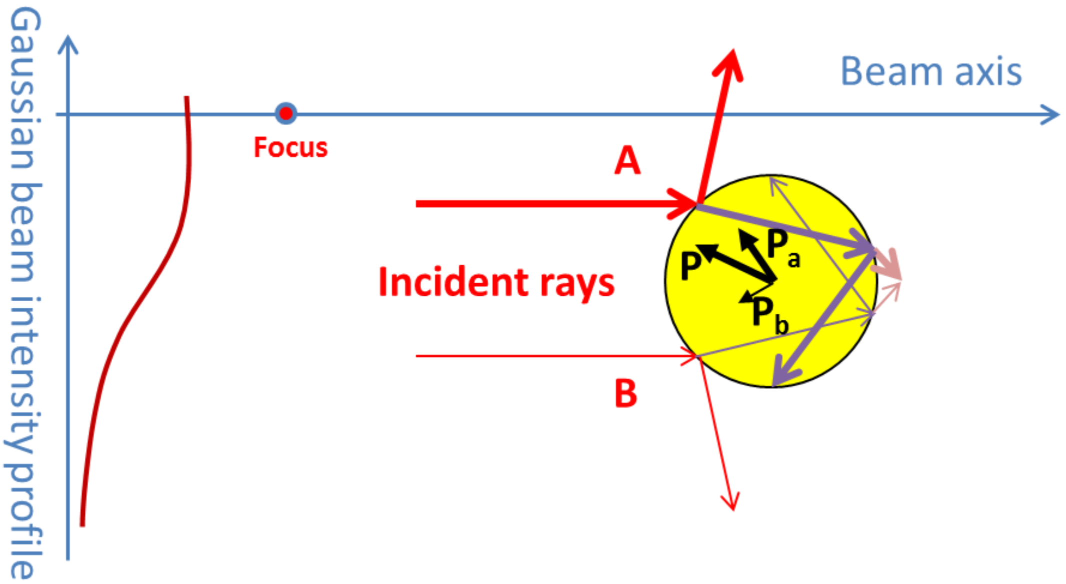

Supplementary Figure 1. Working mechanism of SBAT. A SBAT, with a Gaussian beam intensity profile, emits two representative rays (A and B) along with its scattered rays on the target. Note that the thicker arrow carries higher energy. As the incident rays propagate through the target, the outgoing rays would induce the pressures (P_a and P_b) by the momentum transfer. Since P_a is higher than P_b, the net pressure P will direct the target towards the focus.

[1] Department of Electrical Engineering, The Hong Kong Polytechnic University, Hong Kong.
[2] NIH Transducer Resource Center and Department of Biomedical Engineering, University of Southern California, Los Angeles, CA 90089, USA.
Correspondence and requests for materials should be addressed to K.H.L. (email: kokokh.lam@polyu.edu.hk).

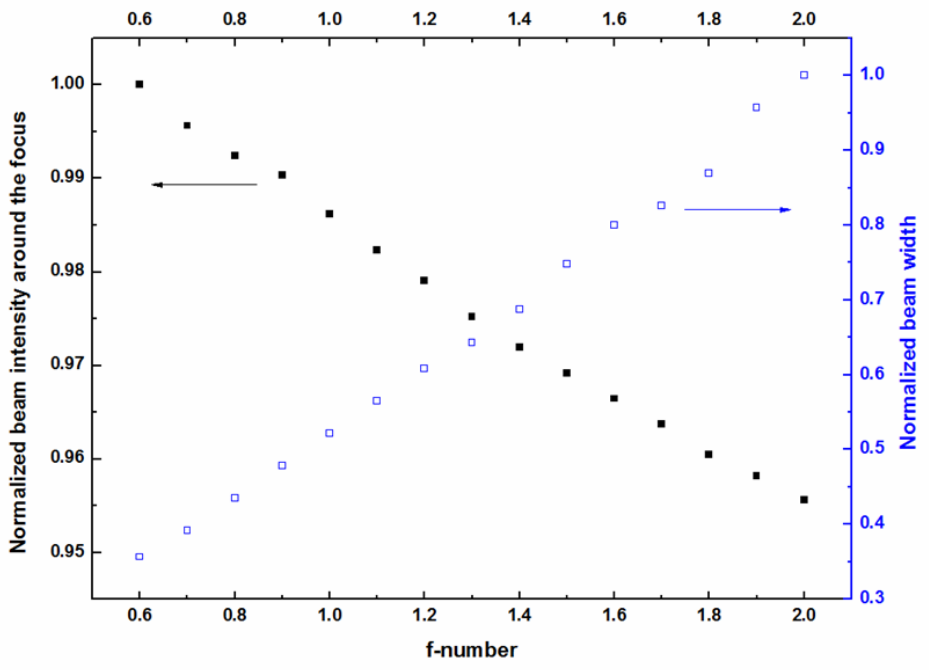

Supplementary Figure 2. Field II simulated results on the beam width and beam intensity around the focus as a function of f-number of the SBAT. The simulation conditions are as follow: Frequency is 60 MHz and the aperture size is 1.6 mm.
